# Lung ultrasound and computed tomography to monitor COVID-19 pneumonia in critically ill patients: a two-center prospective cohort study

**DOI:** 10.1186/s40635-020-00367-3

**Published:** 2021-01-25

**Authors:** Micah L. A. Heldeweg, Jorge E. Lopez Matta, Mark E. Haaksma, Jasper M. Smit, Carlos V. Elzo Kraemer, Harm-Jan de Grooth, Evert de Jonge, Lilian J. Meijboom, Leo M. A. Heunks, David J. van Westerloo, Pieter R. Tuinman

**Affiliations:** 1 Department of Intensive Care Medicine, Amsterdam University Medical Centers, location VUmc, Amsterdam, The Netherlands; 2 Amsterdam Cardiovascular Sciences Research Institute, Amsterdam UMC, Amsterdam, The Netherlands; 3 Department of Intensive Care Medicine, Leiden University Medical Center, Leiden, The Netherlands; 4 Amsterdam Leiden Intensive Care Focused Echography (ALIFE), Amsterdam, The Netherlands; 5 Department of Radiology and Nuclear Medicine, Amsterdam University Medical Centers, location VUmc, Amsterdam, The Netherlands; 6 VU University Medical Center Amsterdam, Postbox 7507, 1007 MB Amsterdam, The Netherlands

**Keywords:** Ultrasonography, Lung, COVID-19, Pneumonia, Critical illness, Respiratory distress syndrome, Adult

## Abstract

**Background:**

Lung ultrasound can adequately monitor disease severity in pneumonia and acute respiratory distress syndrome. We hypothesize lung ultrasound can adequately monitor COVID-19 pneumonia in critically ill patients.

**Methods:**

Adult patients with COVID-19 pneumonia admitted to the intensive care unit of two academic hospitals who underwent a 12-zone lung ultrasound and a chest CT examination were included. Baseline characteristics, and outcomes including composite endpoint death or ICU stay > 30 days were recorded. Lung ultrasound and CT images were quantified as a lung ultrasound score involvement index (LUSI) and CT severity involvement index (CTSI). Primary outcome was the correlation, agreement, and concordance between LUSI and CTSI. Secondary outcome was the association of LUSI and CTSI with the composite endpoints.

**Results:**

We included 55 ultrasound examinations in 34 patients, which were 88% were male, with a mean age of 63 years and mean P/F ratio of 151. The correlation between LUSI and CTSI was strong (*r* = 0.795), with an overall 15% bias, and limits of agreement ranging − 40 to 9.7. Concordance between changes in sequentially measured LUSI and CTSI was 81%. In the univariate model, high involvement on LUSI and CTSI were associated with a composite endpoint. In the multivariate model, LUSI was the only remaining independent predictor.

**Conclusions:**

Lung ultrasound can be used as an alternative for chest CT in monitoring COVID-19 pneumonia in critically ill patients as it can quantify pulmonary involvement, register changes over the course of the disease, and predict death or ICU stay > 30 days.

*Trial registration*: NTR, NL8584. Registered 01 May 2020—retrospectively registered, https://www.trialregister.nl/trial/8584

## Background

Coronavirus disease 2019 (COVID-19) is currently challenging the flexibility and capacity of health care systems around the globe. Five percent of COVID-19 patients are severely ill and require admission to an intensive care unit (ICU), posing an extraordinary challenge to these departments [1]. Resources are stretched thin and require novel solutions on an organizational and medical level [2]. Maximizing critical care capacity by ensuring efficient use of health care workers, devices, personal protective equipment, and other resources is crucial to minimize COVID-19 related death, even in high-income areas [3].

The COVID-19 pneumonia diagnosis is made through laboratory confirmation combined with clinical (or radiological) suspicion. International (and clinical) guidelines recommend the use of computed tomography (CT) for the (repeated) evaluation of COVID-19 pneumonia lung involvement, in particular in case of non-resolving or worsening clinical picture [4]. Semi-quantitative CT scoring systems, such as the severity score (CT-SS), are used to adequately distinguish mild from severe disease [5, 6]. However, the ICU population is severely ill by definition and scanning requires transportation further increasing demands on frail patients and resource-constrained hospitals. Transport outside of isolation carries risks for patients, health care workers, bystanders and requires both contingency plans and post-transport decontamination [7]. The need for repeated scans can be minimized by increasing use of bedside monitoring tools.

Lung ultrasound is superior to standard chest radiography and similar to chest CT for the evaluation of pneumonia and adult respiratory distress syndrome (ARDS) with added benefit of repeatability, low cost, absence of radiation exposure, and ease of use [8, 9]. Several editorials have recommended increasing the use of lung ultrasound during the current pandemic [10–13], but data on its value in diagnosing and especially monitoring COVID-19 pneumonia is still limited [14–16].

We aim to evaluate lung ultrasound as an alternative to CT for monitoring COVID-19 pneumonia lung involvement on the ICU, thereby potentially reducing the need for CT scanning, its associated risks, and costs. Our hypothesis is that the semi-quantitative Lung Ultrasound Score (LUS) strongly correlates with CT-SS (*r* > 0.70), is reactive to clinical evolution, and predicts outcomes similarly in critically ill COVID-19 pneumonia patients.

## Methods

### Study aim, design, and setting

Our aim was to evaluate lung ultrasound as an alternative to CT for monitoring COVID-19 pneumonia lung involvement on the ICU. We conducted a prospective observational cohort study of laboratory-confirmed COVID-19 cases in two academic adult ICUs (Amsterdam UMC, location VUmc, the Netherlands and LUMC, Leiden, the Netherlands). Bedside ultrasound evaluations are regularly performed in these centers, provided there is a relevant clinical indication and an available certified ultrasound physician. The local ethics boards approved the study and usage of data gathered during routine ultrasound without informed consent. This trial was registered in Dutch Trial Registry (ID: NL8584) and was drafted in compliance with the STROBE guidelines [17].

### Participants and outcome variables

Adult (> 18 years) patients admitted to the ICU and diagnosed with COVID-19 between April 1st and May 30th were screened. They were included when a clinically indicated 12-zone lung ultrasound was performed by an available clinician (convenience sampling) and recorded within 48 h of a chest CT-scan. Baseline characteristics (age, sex, height, weight), ventilator settings, arterial blood gas values, and Sequential Organ Failure Assessment score (SOFA) were collected from the electronic patient database as close to time of CT as possible. The ratio of arterial oxygen partial pressure to fractional inspired oxygen (P/F ratio) was calculated based on arterial blood gas values and concurrent ventilator oxygen setting. We used the Kigali Modification of the Berlin Definition of ARDS (so non-ventilated patients could also be classified) to classify COVID-19 cases as mild, moderate, and severe [18]. P/F ratio for non-ventilated patients on low-flow oxygen was estimated using an established conversion method [19]. Follow-up started at intubation or, for non-ventilated patients, at ICU admission. Patients were followed for the longest possible follow-up until discharge, death, or, when still admitted, until drafting of this manuscript. An inclusive composite outcome of death or ICU stay > 30 days was calculated.

### Lung ultrasound

Images were acquired or supervised by certified clinicians (*n* = 8) using the Sonosite-EDGE II or Philips Lumify ultrasound system. Certification entailed a 2-day course and thereafter supervision by a physician with extensive ultrasound experience (> 5 years) until sufficient expertise was reached (a minimum of 30 exams) prior to this study [20]. All measurements were performed on supine patients using a 10–5 MHz linear transducer (VUmc) or a Lumify 4-1MHZ MHz S4-1 broadband phased array transducer (LUMC) with the lung examination setting with a depth of > 6 cm [21]. Measurements were conducted according to the 12-zone LUS protocol: one superior and inferior zone on anterior, lateral, and posterior areas of each hemithorax [22]. Offline analyses of ultrasound images were performed by researchers blinded to the patient’s CT results. The offline reviewers determined the semi-quantitative LUS of involvement: normal = 0, well-separated B-lines = 1; coalescent B-lines, small consolidation or pleural effusion (< 1 cm) = 2, consolidation, large consolidation or pleural effusion (> 1 cm) = 3; of each zone [22]. A global score was calculated by summing the scores of all 12 lung regions, ranging from 0 (i.e., all zones with normal aeration) to 36 (i.e., all zones with large consolidation or large quad signs). Regional scores were calculated by summing the field scores of anterior, lateral, and posterior regions (ranging from 0 to 12) or superior and inferior regions (ranging from 0 to 18). An antero-1-lateral score (3 views per hemithorax) was derived by summing the anterior and lateral scores without the anteroinferior points [23]. Missing scores values from one or more regions that were non-examinable were resolved by expressing the lung ultrasound score as an ‘involvement index’ (LUSI): (actual score/total score achievable) × 100. The number of potential regions was at most 12, 6, or 4 for the LUSI and regional scores, respectively. As such, an involvement percentage of 0% would represent normal aeration on all lung fields and a score of 100% would represent consolidation on all lung fields.

### Chest computed tomography

Chest CT was performed on two multidetector CT scanners: Siemens Somatom Drive (Siemens Healthineers, Erlangen, Germany), and a GE Discovery 750 HD (GE Healthcare, Milwaukee, MI). All patients underwent CT scanning of the chest in the supine position during end-inspiration. Slice thickness for all scanners was between 0.625 and 1.25 mm. HD lung (GE Healthcare) kernel, pulmonary Br59F kernel (Siemens Healthineers) were applied. The chest CT scans were performed on clinical indication (diagnosis, non-resolving-, or worsening clinical picture) and evaluated by a radiologist blinded for lung ultrasound results. The radiologists in the Netherlands determined a CT-SS based on a previously validated study in severe acute respiratory syndrome [24]. The five lobes of the lung were each scored for involvement with ground glass or consolidation: 0% (0 points), 1–5% (1 point), 5–25% (2 points), 25–50% (3 points), 50–75% (4 points), or > 75% (5 points). Data on the CT-SS, ranging from 0 to 25, was collected from the radiology report. A CT-SS ‘involvement index’ (CTSI), with 0% representing no involvement, and 100% representing > 75% involvement on all five lobes, was also calculated for the CT-SS (CTSI).

### Statistical analysis

Statistical analyses were performed using SPSS IBM version 22 (SPSS Inc., Chicago, IL, USA) and the *R* language for statistical computing with the *tidyverse* suite of packages [25]. Demographic, clinical, and outcome variables were presented as means ± standard deviations (± SD), medians and interquartile range [IQR], or numbers (percent %) when appropriate. A Shapiro–Wilk’s test, visual inspection of histograms, and Q–Q plots were used to determine data distribution.

### Baseline and different zones

An ANOVA one-way (or Kruskal–Wallis if non-parametric) test was used to compare baseline characteristics across categories of ARDS severity. The same test was used to determine whether there were differences in (regional) LUSI, CTSI, and across ARDS severity categories.

### Primary outcome: correlation, agreement and concordance

The Spearman’s rank test was used to assess the correlation coefficient (*r*) between LUSI and CTSI on all examinations. We used the same test to assess the correlation between different zone regions of LUSI and CTSI for all examinations and only for unique patients. A correlation coefficient between 0.10 and 0.39 indicates weak, 0.4 and 0.69 moderate, and 0.70 and 0.89 a strong positive relationship [26]. A Bland–Altman plot was created to assess agreement. The change in LUSI was assessed by correlating the difference (Δ) of sequential LUSI and CTSI examinations with a Spearman’s rank test. The overall concordance was assessed by allocating full concordance (1) to changes in the same direction, discordance (0) to changes opposite directions, or tie (0.5) when either LUSI of CTSI did not change.

### Secondary outcome: prediction of outcomes

A logistic regression analysis was performed to assess the prediction of LUSI and CTSI on the outcomes of all unique patients. Five independent variables were selected as candidate predictors: age, P/F ratio, SOFA score, LUSI, and CTSI. As LUSI and CTSI are percentages of lung involvement and not strictly continuous variables, they were dichotomized to high involvement (≥ 50%) and low involvement, reflecting the ‘severe illness’ category in the National Institutes of Health guidelines for the management of COVID-19 [27]. A univariate analysis was made for death, ICU stay > 30 days, and their composite. A multivariate analysis was performed for the composite outcome.

### Sample size

A previous study that correlated CT tissue density with LUS for ARDS found a strong correlation coefficient of 0.79 [28]. Considering a two-sided *α* of 0.05 and a *β* of 0.05 this study would require a sample size of 14 to determine that the correlation coefficient differs from zero [29]. Cases were collected until a sufficient sample for clinical evolution was also reached.

## Results

### Patients

**Fig. 1:**
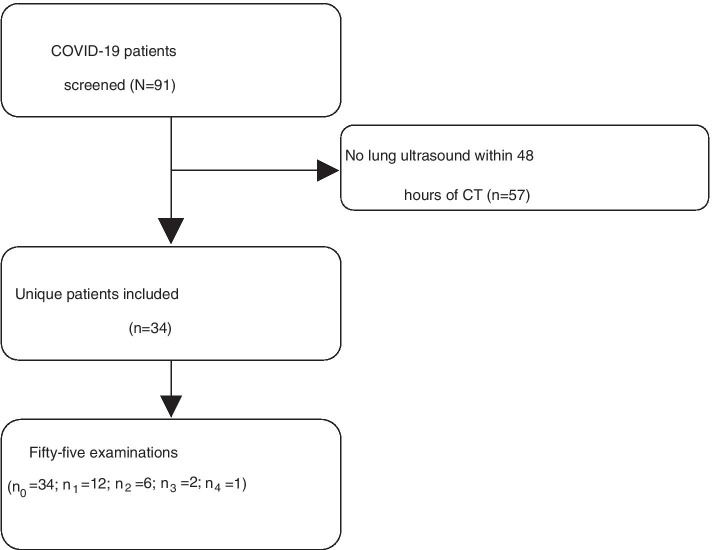
Flowchart of COVID-19 patient screening and inclusion. Legend: *n*_0_ refers to the amount of baseline examinations. *n*_1_, *n*_2_, etc., refers to second examination, third examination, etc., respectively

Out of 91 screened patients, 34 were included with 55 lung ultrasound examinations (a total of 660 zones). Six (0.9%) of these zones were missing (Fig. 1). The median time between the lung ultrasound and CT examination was 17.2 [25.6] hours. Baseline characteristics are shown in Table 1, ventilator settings and arterial blood gas values are shown in Additional file 1: Table S1. The overall patient population was 88% male, with a mean age of 63 (± 10.2), and a BMI of 28.2 (± 4.4). Two patients were not mechanically ventilated at the time of CT and its associated ultrasound examination. The mean follow-up from intubation was 31.8 (± 16.5) days, whereas the mean days from intubation to first follow-up CT after diagnosis was 14.6 (± 10.1).

**Table 1:** Baseline characteristics at time of computed tomography and outcomes

	Overall (*N* = 34)	Mild (*n* = 6)	Moderate (*n* = 25)	Severe (*n* = 3)	*p*-value
P/F ratio	150.3 ± 43.9	222.8 ± 16.1	141.8 ± 20.8	75.6 ± 29.5	
Baseline demographics at CT					
Age (years)	63.0 ± 10.2	61.8 ± 9.0	61.9 ± 10.4	74.0 ± 4.4	0.068
Gender (male)	30 (88.2%)	5 (83.3%)	22 (88.0%)	3 (100%)	0.763
BMI, m/kg^2^	28.2 ± 4.4	29.0 ± 3.5	27.6 ± 4.0	32.1 ± 8.3	0.473
Time since symptoms (days)	23.4 ± 11.8	27.3 ± 14.4	23.7 ± 11.1	13.8 ± 11.4	0.339
SOFA score	7.5 ± 3.4	8.5 [6]	7.4 ± 3.5	7.0 ± 4.6	0.755
CTSI	73.1 ± 18.7	70.7 ± 33.7	73.3 ± 15.2	76.0 ± 12.0	0.930
LUSI	57.6 ± 16.8	53.7 ± 21.8	58.9 ± 16.3	53.7 ± 13.1	0.779
Clinical outcomes					
Discharge (%)	17 (51.5%)	5 (83.3%)	11 (45.8%)	1 (33.3%)	0.636
Still admitted on ICU (%)	7 (21.2%)	1 (16.7%)	5 (20.8%)	1 (33.3%)	0.636
Lost to follow-up (%)	3 (8.8%)	0 (0%)	3 (12.0%)	0 (0%)	0.636
Deceased (%)	6 (17.6%)	0 (0%)	5 (20.0%)	1 (33.3%)	0.636
ICU admission > 30 days (%)	16 (59.4%)	3 (50.0%)	12 (63.2%)	1 (50.0%)	0.817
Composite outcome (%)	22 (66.7%)	3 (50%)	17 (70.8%)	2 (66.7%)	0.626

Variables were presented as means ± standard deviations (± SD), medians and interquartile range [IQR], or numbers (percent %) depending on distribution

*BMI* body mass index, *CT* chest tomography, *CTSI* chest computed tomography severity involvement index, *ICU* intensive care unit, *LUSI* lung ultrasound score involvement, *P/F* ratio between partial oxygen pressure and fraction of inspired oxygen

### Primary outcome: correlation, agreement, and concordance between LUSI and CTSI

**Fig. 2:**
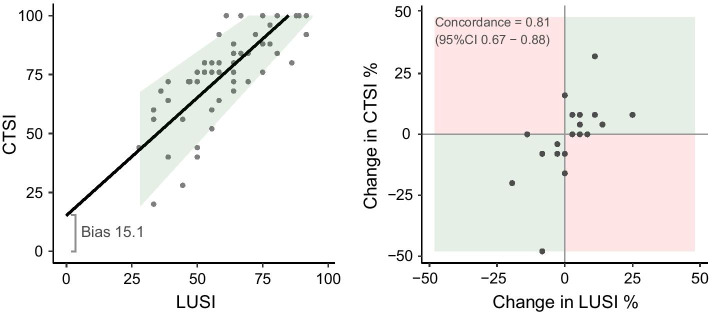
**a** The correlation between LUSI and CTSI. Legend: the shaded area is a Bland–Altman where the line represents the biased association between the measurements (lung ultrasound underestimates CT involvement) and the shaded area represents the limits of agreement. The measurement error decreases with increased involvement. *LUSI* lung ultrasound score severity index; *CTSI* computed tomography severity involvement index. **b** Concordance between changes in sequentially measured LUSI and changes in CTSI. Legend: green squares denote concordance (involvement changes in the same direction) and red squares represent discordance (involvement changes in different directions). *LUSI* lung ultrasound score severity index; *CTSI* computed tomography severity involvement index

The mean LUSI was 58 (± 17) and mean CTSI was 73 (± 19). The correlation between LUSI and CTSI was 0.794 (95%CI 0.67; 0.87). The correlation between LUSI and CTSI is shown in Fig. 2a. The bias was − 15.1 (95%CI − 18.6; − 11.7), indicating that LUSI underestimated CTSI by 15.1%, and the limits of agreement ranged from − 40 to 9.7 (Additional file 2: Figure S1). The slope of the association between LUSI and CTSI (0.87, 95%CI 0.65–1.10) was not significantly different from 1, indicating that the magnitude of the bias was not related to the magnitude of involvement (there was no proportional bias). The mean measurement error decreased with involvement (*p* = 0.007), indicating that LUSI reflected CTSI more precisely with higher involvement.

Twenty-one follow-up examinations (lung ultrasound and CT) were performed in 12 unique patients. LUSI change had a strong correlation with CTSI change (*r* = 0.748, *p* < 0.001). The concordance between changes in sequentially measured LUSI versus changes in CTSI was 0.81 (95%CI 0.67–0.88). There were no absolute discordant measurements (increased involvement on LUS but decreased involvement on CT or vice versa) (Fig. 2b).

LUSI was different across regional zones (*p* < 0.001–0.018), with involvement being the least in the anterior zones, moderate in the lateral zones, and most in the posterior zones (Additional file 1: Table S2). All of the distinct regional LUSI correlated with the CTSI (Additional file 1: Table S3). The antero-1-lateral LUSI (one anterosuperior zone and two lateral zones) had the highest correlation coefficient with CTSI (*r* = 0.81).

### Secondary outcome: LUSI, CTSI, and clinical outcomes

**Fig. 3:**
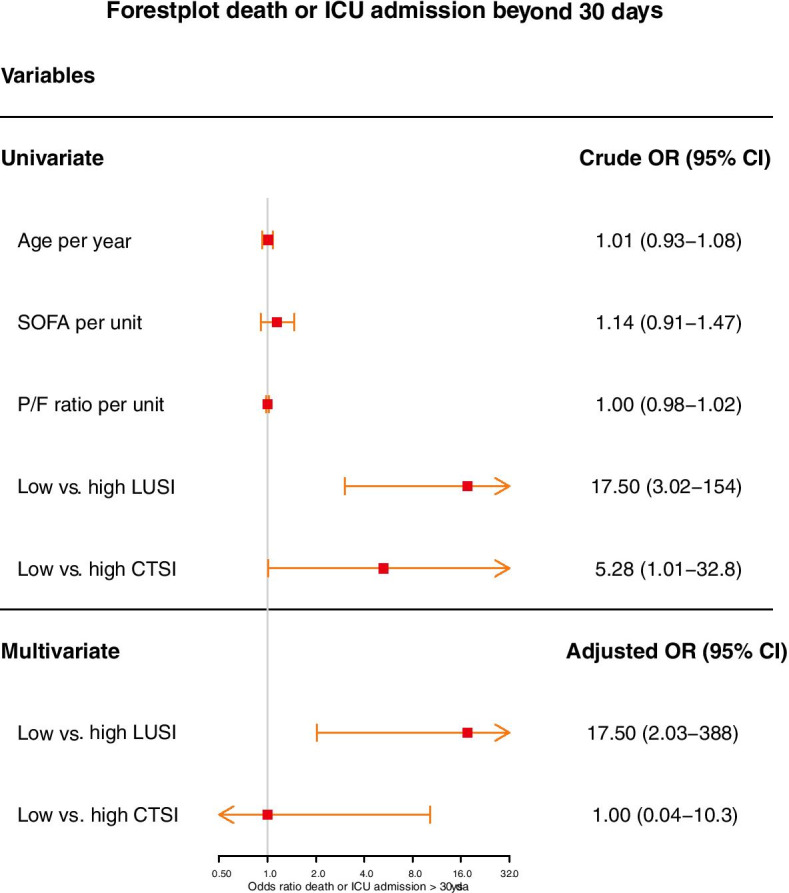
Forest plot of OR for the composite outcome of death or ICU stay > 30 days. Legend: high lung ultrasound score involvement index (LUSI) was ≥ 50% whereas high computed tomography severity involvement index (CTSI) was ≥ 65%. Only statistically significant variables were included in the multivariate model. *OR* odds ratio; *ICU* intensive care unit

The univariate analyses for death and ICU stay > 30 days are shown in Table 2. LUSI and CTSI at the first CT were higher in patients with an ICU stay > 30 days. With only 6 deaths (17.6%) and 7 patients still admitted (21.1%), neither the involvement on imaging, nor the baseline characteristics were associated with death. A composite outcome of death and ICU stay > 30 days could be created for 33 (97.1%) of patients. Only LUSI (OR 17.5; 95%CI 3.02–154) and CTSI (OR 5.28; 95%CI 1.01–32.8) predicted the composite outcome in the univariate analysis. Lung ultrasound involvement at the antero-1-lateral zone had comparable association with composite outcome as LUSI or CTSI (OR 16.9; 95%CI 3.15–124), but was not included in multivariate model to avoid overfitting. The multivariate analysis only retained LUSI for the prediction of composite outcome (OR 17.5; 95%CI 2.03–388.7) (Fig. 3).

**Table 2:** Univariate analysis of predictors and outcomes

	Death (*n* = 24)			Length of ICU stay (*n* = 27)		
	No (*n* = 18)	Yes (*n* = 6)	OR (95% CI)	< 30 (*n* = 11)	> 30 (*n* = 16)	OR (95% CI)
LUSI						
Low	8 (89%)	1 (11%)	Ref	7 (87%)	1 (13%)	Ref
High	10 (67%)	5 (33%)	4.00 (0.50–85.1)	4 (21%)	15 (79%)	26.3 (3.37–575)*
Antero-1-lateral LUSI						
Low	9 (90%)	1 (10%)	Ref	8 (80%)	2 (20%)	Ref
High	9 (64%)	5 (36%)	5.00 (0.63–106)	3 (18%)	14 (82%)	18.7 (3.01–180)*
CTSI						
Low	5 (71%)	2 (29%)	Ref	5 (83%)	1 (17%)	Ref
High	13 (76%)	4 (24%)	0.77 (0.11–6.82)	6 (29%)	15 (71%)	12.5 (1.59–268)*
P/F ratio per unit	160 ± 53	136 ± 23	0.99 (0.96–1.01)	151 ± 61	157 ± 43	1.00 (0.99–1.02)
SOFA Score per unit	6.9 ± 3.6	8.5 ± 3.0	1.14 (0.87–1.53)	5.1 ± 2.6	7.9 ± 3.5	1.12 (0.89–1.45)
Age per year	63 ± 8	67 ± 8	1.07 (0.95–1.25)	62 ± 8	61 ± 12	0.99 (0.91–1.07)

Variables were presented as means ± standard deviations (± SD), or numbers (percent %) depending on distribution. Involvement ≥ 50% was the cutoff for LUSI and antero-1-lateral LUSI, whereas involvement ≥ 65% was the cutoff for CTSI. Odds ratios with significant (*p* < 0.05) associations are indicated by *

*CI* confidence interval, *CTSI* chest computed tomography severity involvement index, *ICU* intensive care unit, *LUSI* lung ultrasound score involvement, *OR* odds ratio, *P/F* ratio between partial oxygen pressure and fraction of inspired oxygen, *SOFA* sequential organ failure assessment

The cutoff for low-to-high involvement of CTSI was adjusted with the underestimation bias (15%) to ≥ 65%. This produced a larger odds ratio with a more narrow confidence interval than the original ≥ 50% cutoff in all analyses.

## Discussion

The main findings of this study on lung ultrasound and CT for monitoring COVID-19 pneumonia in critically ill patients were the following: (1) LUSI has a strong correlation with CTSI. A six-zone LUSI performs similar to a twelve-zone LUSI; (2) LUSI is reactive to change in CTSI in sequential examinations (concordance = 81%); (3) high pulmonary involvement on LUSI and CTSI predicts ICU length of stay, but not death. Only LUSI was retained in the multivariate regression model to predict the composite of death and ICU stay > 30 days.

Our results suggest that lung ultrasound can be used as a substitute of chest CT for the monitoring of COVID-19 pneumonia severity in critically ill patients. Lung ultrasound underestimates CT involvement by 15.1% but the clinical relevance of this bias seems limited. It could be attributed to lung ultrasound’s inability to identify pathology beyond the pleural line, inadvertently missing centrally located consolidation [10], although COVID-19 pneumoniae all present with a peripheral or mixed distribution [30]. Another reason might be CTSS’ skewed scoring system which does not discriminate pulmonary involvement above 75%. Although we used CT as reference standard, a recent study suggests that lung ultrasound provides a higher sensitivity than CT to detect pulmonary content variations in COVID-19 [31].

In addition, lung ultrasound was able to concordantly detect clinical evolution of lung involvement, demonstrating it can evaluate the course of COVID-19 pneumonia over time similarly to chest CT. It has been shown that chest CT identifies progression of COVID-19 pneumonia from ground-glass opacities towards consolidations and subsequent absorption [32]. This is important as there appears to be a time-related disease spectrum for COVID-19, with different respiratory treatments for different phenotypes [33]. The non-ARDS type is characterized by mainly ground-glass densities on CT and a low amount of non-aerated tissue, indicating minimal recruitability. On the other hand, the ARDS type shows a remarkable increase in non-aerated tissues, increasing the potential for recruitability [34].

Increased pulmonary involvement might indicate a protracted ICU stay. In line with this, our study found that high pulmonary involvement on lung ultrasound (≥ 50%) and CT (≥ 65%) carried increased risk of ICU stay beyond 30 days. A restricted multivariate model showed that high involvement on lung ultrasound carried the best prediction for outcomes. It is important to note that COVID-19 is not merely a pneumonia, and many of its patient-centered outcomes also depend on complications [35]. Major COVID-19 complications often encountered on the ICU are pulmonary embolism or (fungal) superinfections, which do require CT angiography or bronchoalveolar lavage for the respective diagnosis.

The use of lung ultrasound as the primary monitoring modality potentially reduces the number of medical devices used, thus reducing costs, fomites, and patient transportation, and sparing personal protective equipment as well as (time of) health care workers [36]. This is important since COVID-19 is capable of rapid nosocomial spread through fomites. This is highlighted by a South African report where a single introduction spread through five hospital wards and 135 patients mainly through indirect contact with (medical) equipment [37]. Concurrently, ultrasound can also be employed on the ICU for multiple indications surrounding a COVID-19 admission, such as diagnostic or procedural guidance [38–40]. Moreover, our data suggests that a six-zone lung ultrasound examination performs similar to a twelve-zone examination. This result needs to be validated in another study but does coincide somewhat with previous literature and the widely used BLUE profile [23, 41].

In summary, both lung ultrasound and CT are equally capable of evaluating pulmonary involvement and registering changes over the course of disease using semi-quantitative visual scoring systems. Pulmonary involvement on lung ultrasound shows a stronger association with death and ICU stay > 30 days. These results suggest that CT can be reserved for those situations where lung ultrasound does not adequately explain the clinical question, for example when a pulmonary embolism is suspected.

### Limitations and strengths

Our study had a sufficiently large sample size to make an inference about the primary outcome, but was not powered for the secondary outcome. The estimated odds ratio shows a definite association with outcome, but its confidence intervals were large. This can be explained by the low absolute occurrence of outcome events. Time between scan and ultrasound was rather large (48 h). Based on clinical observations of the course of COVID-19 pneumonia severity it is unlikely that drastic changes in pulmonary involvement occur within 2 days. However, changes in volume status or ventilator settings might have conferred some non-selective changes. This study was not designed to show clinical equivalence of these two imaging techniques, but the correlation of respective validated semi-quantitative scoring systems for pulmonary involvement. Future studies might investigate the use of (semi-)automated quantification methods for the evaluation of pulmonary involvement [42, 43]. Patient deterioration caused by pulmonary embolism is especially relevant considering the thrombogenicity found in COVID-19 disease [44]. It is likely that bedside ultrasound can be employed to identify peripheral deep venous thrombosis or other ultrasonographic signs of pulmonary embolism, but no such investigation exists in COVID-19 population and speculation therefore lies beyond the scope of this study [45, 46].

Our study has several strengths. Although the manuscript was submitted before definite end points were reached in all patients, we created a functional outcome for 97.1% of patients with the composite ICU outcome. We investigated the correlation between lung ultrasound and CT using multiple ultrasound operators and two centers, increasing generalizability and validity of the results. This is especially relevant considering global spread of COVID-19 and the implications of these results for COVID-19 monitoring in ICUs worldwide.

## Conclusion

This two-center prospective cohort study shows that there is good correlation and agreement between lung ultrasound and CT based scoring systems for evaluating pulmonary involvement in patients with COVID-19 pneumonia. Lung ultrasound can quantify, detect changes in, and prognosticate pulmonary involvement. Considering the drawbacks of CT scanning and patient transportation our results support the increased uptake of lung ultrasound during the COVID-19 pandemic.

## Supplementary Information

**Additional file 1: Table S1.** Ventilator settings and arterial blood gas values: overall and across ARDS categories. **Table S2.** Distribution of lung ultrasound involvement in regions: overall and across ARDS categories. **Table S3.** Correlation between regional lung ultrasound involvement index and CT for examinations and patients.

**Additional file 2: Figure S1.** Bland–Altman plot. The bias was − 15.1 (95%CI − 18.6; − 11.7), indicating that LUSI underestimated CTSI by 15.1%.

## Data Availability

The datasets used and/or analyzed during the current study are available from the corresponding author on reasonable request.

## Abbreviations

ARDS: Acute respiratory distress syndrome
CI: Confidence interval
COVID-19: Coronavirus Disease 2019
CT: (Chest) computed tomography
CTSI: CT severity involvement index
CT-SS: Computed Tomography Severity Score
FiO2: Fraction of inspired oxygen
ICU: Intensive care unit
IQR: Inter quartile range
LUS: Lung Ultrasound Score
LUSI: Lung ultrasound score involvement index
PaO2: Partial oxygen pressure
P/F ratio: Ratio between partial oxygen pressure and fraction of inspired oxygen
SD: Standard deviation
SOFA: Sequential organ failure assessment
