# Supplementary material for: Lung ultrasound and computed tomography to monitor COVID-19 pneumonia in critically ill patients: a two-center prospective cohort study
Source: Intensive Care Med Exp. 2021 Jan 25;9:1. doi: 10.1186/s40635-020-00367-3 (PMC7829056; doi:10.1186/s40635-020-00367-3)
Supplement: Supplementary file 1 — Additional file 1: Table S1. Ventilator settings and arterial blood gas values: overall and across ARDS categories. Table S2. Distribution of lung ultrasound involvement in regions: overall and across ARDS categories. Table S3. Correlation between regional lung ultrasound involvement index and CT for examinations and patients. [file 40635_2020_367_MOESM1_ESM.docx]

**Additional Material**

**Additional Material table S1.** Ventilator settings and arterial blood gas values: overall and across ARDS categories.

|  | Overall | Mild | Moderate | Severe | P-value |
| --- | --- | --- | --- | --- | --- |
| Ventilator settings |  |  |  |  |  |
| Controlled mode (%) | 19 (63.3%) | 3 (75.0%) | 14 (58.3%) | 2 (100.0%) | 0.785 |
| FiO2 (%) | **52.7±13.0** | **39.4±3.8** | **52.2±9.5** | **79.0±11.5** | **0.001*** |
| PEEP (cmH2O) | 9.8±2.7 | 9.0±2.5 | 9.6±1.8 | 14.6±7.9 | 0.610 |
| Maximum pressure (cmH2O) | 22.4±5.6 | 22.6±10.6 | 22.1±4.1 | 25.8±12.4 | 0.971 |
| Minute volume (L/min) | 10.0[5.4] | 9.9[4.2] | 10.7[5.3] | 8.5 (8.1-8.9) | 0.270 |
| Tidal volume (ml/kg BW) | 4.99[1.57] | 4.11[2.02] | 5.15[1.34] | - | 0.077 |
| Arterial blood gas values |  |  |  |  |  |
| pH | 7.37±0.09 | 7.36±0.07 | 7.37±0.09 | 7.42 (7.32-7.51) | 0.556 |
| pCO2 (kPa) | 6.8[2.25] | 7.0[4.6] | 7.2[1.8] | 7.8 (4.3-13.5) | 0.954 |
| pO2 (kPa) | **9.7[2.3]** | **11.9[1.8]** | **9.5[2.0]** | **9.3(3.2-9.4)** | **0.011*** |

^BW body weight; P/F ratio between partial oxygen pressure and fraction of inspired oxygen; FiO2 fraction of inspired oxygen; pO2 partial oxygen pressure (arterial). variables were presented as means ± standard deviations (±SD), medians and interquartile range [IQR], or numbers (percent %) depending on distribution. Differences with a p-value below 0.05 are indicated by *.^

**Additional Material figure S1.** Bland-Altman plot. The bias was -15.1 (95%CI -18.6; -11.7), indicating that LUSI underestimated CTSI by 15.1%

**Additional Material table S2.** Distribution of lung ultrasound involvement in regions: overall and across ARDS categories.

|  | Overall (N=55) | Mild (N=12) | Moderate (N=37) | Severe (N=6) | - P-value |
| --- | --- | --- | --- | --- | --- |
| Anterior LUSI | 45.3±19.8 | 39.6±19.2 | 48.6±19.4 | 36.1±21.5 | 0.223 |
| Lateral LUSI | 63.3±18.9 | 59.0±24.2 | 64.8±17.9 | 62.5±13.7 | 0.714 |
| Posterior LUSI | 73.0±19.6 | 70.8±17.6 | 73.2±21.2 | 76.4±15.3 | 0.816 |
| Superior LUSI | 58.3±18.4 | 54.6±20.8 | 59.8±18.6 | 57.4±13.0 | 0.628 |
| Inferior LUSI | 62.2±16.7 | 58.3±18.1 | 63.9±17.0 | 59.3±12.5 | 0.578 |
| **↓** P-value | 0.000* | 0.017* | 0.000* | 0.018* | - |

^LUSI lung ultrasound score involvement; P/F ratio between partial oxygen pressure and fraction of inspired oxygen; variables were presented as means ± standard deviations (±SD). The p-value at the bottom of the columns corresponds to the difference between the regional variants of LUSI. The p-value at the end of the rows corresponds to the difference between the different P/F categories at different regions. Differences with a p-value below 0.05 are indicated by *.^

**Additional Material table S3.** Correlation between regional lung ultrasound involvement index and CT for examinations and patients.

|  | Unique examinations (N=55) | Unique patients (N=34) |
| --- | --- | --- |
| LUSI | 0.794 | 0.750 |
| Anterior LUSI | 0.629 | 0.629 |
| Lateral LUSI | 0.722 | 0.745 |
| Posterior LUSI | 0.592 | 0.583 |
| Superior LUSI | 0.709 | 0.679 |
| Inferior LUSI | 0.715 | 0.702 |
| Anterior-1-lateral LUSI | 0.809 | 0.815 |

^LUSI lung ultrasound score involvement; all values were correlation coefficients (r); all values were associated with a p-value below 0.05.^
